# A Patient Survey Exploring the Burden of Inflammatory Back Pain in Patients With Known Psoriasis

**DOI:** 10.7759/cureus.51968

**Published:** 2024-01-09

**Authors:** Sophia Watts, Lydia G Nagib, Sian Bamford, Adil Sheraz, Hasan Tahir

**Affiliations:** 1 Dermatology, Royal Free London NHS Foundation Trust, London, GBR; 2 Medicine, Royal Free London NHS Foundation Trust, London, GBR; 3 Physiotherapy, Royal Free London NHS Foundation Trust, London, GBR; 4 Division of Medicine, University College London, London, GBR

**Keywords:** ankylosing spondylitis, arthritis, inflammatory back pain, axial spondyloarthritis, psoriasis

## Abstract

Background

In the United Kingdom, diagnostic delay remains a challenge in axial spondyloarthritis (axSpA). Psoriasis is a frequently identified extra-musculoskeletal manifestation associated with axSpA. In this study, we aimed to determine the prevalence of inflammatory back pain (IBP) in psoriasis patients at a specialized psoriasis dermatology clinic in a London NHS Trust. Our primary goal was to identify psoriasis patients with IBP who were not referred to a rheumatologist, potentially leading to axSpA diagnostic delays. Additionally, we aimed to investigate factors contributing to these delays and strategies to address them.

Methodology

A patient survey consisting of 22 questions was used to assess the prevalence of IBP among 66 psoriasis patients attending a weekly specialized psoriasis dermatology clinic within a London NHS Trust between May and July 2023. The survey comprised patient demographic information along with inquiries about the existence of back pain exceeding three months. The Berlin Criteria was utilized to identify IBP among patients who reported experiencing back pain for over three months. Additionally, the survey sought information on prior diagnosis of axSpA and whether participants had consulted healthcare professionals regarding their back pain.

Results

Of the 66 patients invited, 51 (77%) completed the survey. The average age of the patients was 50 years (range = 19-74 years), with 58.8% being female. The mean duration of psoriasis was 15.7 years (range = 2-44 years). Overall, 45% (23/51) reported back pain lasting over three months. Among the patients who reported back pain for more than three months, 13 met the Berlin Criteria for IBP (25% of the total surveyed), and only four of these patients had a diagnosis of axSpA. Notably, seven patients (14% of the total surveyed) potentially had undiagnosed axSpA. General practitioners (GPs) were commonly consulted for back pain, yet only 39% of those with prolonged back pain had seen a rheumatologist. Despite experiencing prolonged back pain, 17% of patients had not sought healthcare advice for their symptoms.

Conclusions

This study highlights that IBP is a common yet underdiagnosed comorbidity in psoriasis patients. Dermatologists, GPs, and other allied healthcare professionals play a crucial role in detecting early axSpA. However, limited awareness of IBP hinders its identification in psoriasis patients and subsequent referral to rheumatologists. This highlights the need for improving awareness and education regarding axSpA among dermatologists and allied healthcare professionals as well as the public and patients to ensure timely diagnosis. The development of simple and easy-to-administer screening questionnaires to aid non-rheumatologists in identifying patients with IBP together with simplified referral pathways would increase onward referrals of appropriate patients to rheumatologists.

## Introduction

In the United Kingdom, diagnostic delay remains a challenge in axial spondyloarthritis (axSpA). A comprehensive literature review demonstrated a mean average of 8.7 years of delay between symptom onset and formal diagnosis [1,2]. Numerous studies have demonstrated that early diagnosis and treatment of axSpA are associated with better outcomes [1-3]. Early identification and referral of patients with a high suspicion of axSpA to rheumatology is important as this is often the rate-limiting step in timely diagnosis and treatment [1-3].

Spondyloarthritis (SpA) is a group of overlapping diseases characterised by inflammation in the spine (spondylitis) and joints (arthritis) with symptoms of inflammatory back pain (IBP) [3]. Types of SpA include non-radiographic axSpA, ankylosis spondylitis (AS), psoriatic arthritis (PsA), reactive arthritis and undifferentiated SpA [3].

Arthritis is the most common extracutaneous manifestation of psoriasis [4,5], impacting around 30% of individuals with this condition. PsA manifests as various joint issues (peripheral arthritis, enthesitis, dactylitis) and skin/nail changes [4,5].

In PsA, 20% to 75% of cases involve the axial skeleton (axPsA) [4-6], correlating with severe psoriasis and heightened pain, limiting physical abilities, and reducing the quality of life of patients with this condition compared to those without [7]. Early detection of axPsA is vital to prevent permanent joint and spine damage [7]. Around 9% of axSpA patients also have psoriasis, and skin symptoms usually precede joint problems, allowing dermatologists to screen for IBP before severe joint and spine damage occurs [8,9].

In this study, we aim to investigate the prevalence of IBP in psoriasis patients in a specialist psoriasis dermatology clinic and determine how many of these patients had consulted a rheumatologist and received an axSpA diagnosis, highlighting those who had not seen a rheumatologist and were potentially undiagnosed.

In addition, we aim to explore methods that can be used to aid dermatologists and allied healthcare professionals in identifying signs of IBP requiring referral to rheumatology, as well as ways to streamline the referral process for enhanced ease and efficiency. The primary objective of the study is to enhance the prognosis and quality of life of patients facing this condition in accordance with the National Axial Spondyloarthritis Society Aspiring to Excellence quality improvement initiative [10].

## Materials and methods

A total of 66 patients (18 years and older) with known psoriasis attending a weekly specialist psoriasis clinic within a dermatology department in a London-based NHS Trust between May 2023 and July 2023 (inclusive) were invited to complete a 22-question survey. The aim of the survey was explained, and verbal consent was obtained from participants.

The survey questionnaire was designed by a consultant rheumatologist from the same NHS organisation based on our prior uveitis study [11] and was adapted for psoriasis patients. The questionnaire included information about the patient’s age, duration of psoriasis, and whether they have ever received any biologic agents. Participants were asked if they had any back pain lasting more than three months. If patients reported back pain they were asked about the age of onset of back pain, whether it responded to non-steroidal anti-inflammatory drugs, whether it improved with activity or rest, the location of the back pain, nocturnal awakening due to back pain, and the presence of alternating buttock pain. Patients who reported back pain were asked if they sought advice from a healthcare professional regarding their back pain and were further questioned on which healthcare professional(s) they had consulted. The full questionnaire is detailed in the Appendices.

We chose the Berlin Criteria as a simple screening tool to identify those with IBP, as axial MRI scans and human leukocyte antigen B27 tests are not typically performed in a dermatology clinic [12]. The criteria are back pain experienced for more than three months, under the age of 50, and fulfilling at least two of the following criteria: (1) morning stiffness lasting >30 minutes, (2) improvement in back pain with exercise and not rest, (3) nocturnal awakening during the second half of the night due to pain, and (4) alternating buttock pain.

All patients received the usual standard of care, and ethical clearance/institutional review board approval was not required for this study according to UK law.

We analysed all data derived from the patient survey using descriptive statistics. Frequencies and percentages were reported for categorical data. For continuous variables, means and ranges were calculated.

## Results

A total of 66 patients were approached, and 51 patients completed the questionnaire. The average age of those who completed the questionnaire was 50 years (range = 19-74 years), and 58.8% of those who completed the questionnaire were female. The mean duration of psoriasis was 15.7 years (range = 2-44 years). The number of patients who reported back pain lasting for more than three months was 23 out of 51 (15 females and eight males), i.e., 45% of the total participants in the survey. Only 7.8% of respondents had a known diagnosis of axSpA. Overall, of the 23 participants who reported back pain for more than three months, 13 (seven females and six males) fulfilled the Berlin Criteria [12] for IBP. Four (three males and one female) of those patients had a known diagnosis of axSpA. Of the remaining nine patients fulfilling the Berlin Criteria for IBP, two patients had seen a rheumatologist and seven of the patients (14% of the total surveyed) who had not seen a rheumatologist potentially had undiagnosed axSpA. See Table 1 for detailed demographic and clinical characteristics from the patient survey.

**Table 1:** Detailed demographic and clinical characteristics from our patient survey (n = 51).

Measure	Result
Age on completing the survey in years (mean)	50
Sex, female (%, participants)	58.8%, 30
Length of time with uveitis, years (mean)	15.7 years
Back pain for >3 months (%, participants)	45%, 23
Age of back pain onset, years (mean)	33
Duration of back pain, years (mean)	16
Back pain fulfilling the Berlin Criteria (%, participants)	25%, 13
Known diagnosis of axSpA (%, participants)	7.8%, 4
Concurrent uveitis (%, participants)	25%, 13

Of the 23 participants who reported back pain for more than three months, known AS was reported by 17% (n = 4), known inflammatory bowel disease was reported by 13% (n = 3), and uveitis was reported by 17% (n = 4). From the Berlin Criteria, the most common symptom reported was morning stiffness (60.8%), followed by nocturnal awakening (52%). See Table 2 for a detailed breakdown of Berlin Criteria features.

**Table 2:** Presence of the features of the Berlin Criteria in the 23 participants who reported back pain.

Measure	%, participants
Morning stiffness >30 minutes	60.8%, 14
Waking in the second half of the night with pain	52%, 12
Alternating buttock pain	47.8%, 11
Improvement with activity	30.4%, 7

**Figure 1:**
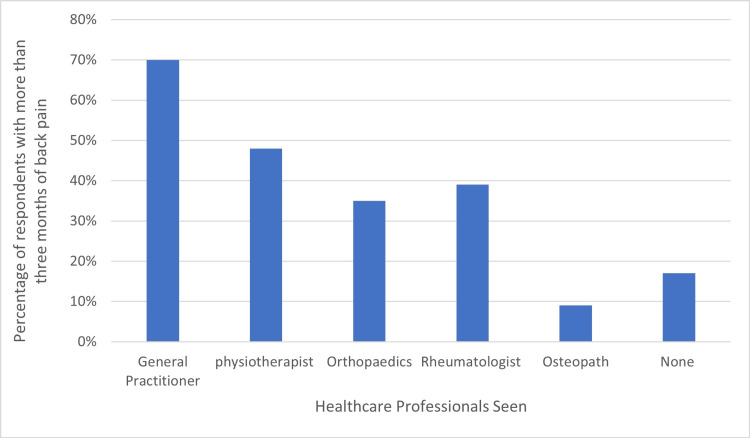
Healthcare professionals seen by patients reporting more than three months of back pain.

Of the 23 patients with back pain for more than three months, 83% (n = 19) had sought advice from a healthcare professional. Respondents had most frequently seen general practitioners (GPs) (70%, n = 16), followed by physiotherapists (48%, n = 11) for their back pain. Only nine (39%) patients had seen a rheumatologist, and 17% (n = 4) had not consulted any healthcare professional despite reporting back pain for more than three months (Figure 1).

## Discussion

Numerous studies have shown that, on average, up to 30% of patients with psoriasis treated at dermatology centres have undiagnosed PsA including those with axPsA that comes under the umbrella of axial axSpA (conditions that typically present with IBP symptoms) [5,13].

Our previous survey of patients with uveitis demonstrated a significant burden of IBP and potentially undiagnosed axSpA [11]. Similarly, within our dermatology clinic, 45% of surveyed psoriasis patients experienced back pain for over three months, and only 17% of them had a confirmed axSpA diagnosis. Our findings echo the prospective multicentre study by Proft et al. where 42.5% of psoriasis patients from dermatology clinics met the criteria for referral to rheumatology (criteria included adults over 18 with chronic back pain lasting over three months and no recent treatment with disease-modifying anti-rheumatic drugs/biologics within 12 weeks before screening) [14].

Of the patients enduring undiagnosed back pain, 68% fulfilled the Berlin Criteria, accounting for 25% of all participants. Among those meeting the Berlin Criteria, only 31% had an established diagnosis of axSpA. Notably, 30% of patients who fulfilled the Berlin Criteria but had not consulted a rheumatologist (14% of the total surveyed) potentially had undiagnosed axSpA. This highlights the significant prevalence of back pain in psoriasis patients at the dermatology clinic and missed opportunities by dermatologists to identify IBP earlier, potentially requiring referral to rheumatologists; approximately one in six psoriasis patients in this clinic may require this referral.

Regarding patients seeking help for their back pain, 83% consulted healthcare professionals, mainly GPs (70%) and physiotherapists (48%). Notably, only 39% of patients were assessed by a rheumatologist. This reveals a significant gap, where GPs and physiotherapists might have missed identifying psoriasis patients needing rheumatology referrals.

As our study results have shown, the lack of referrals to rheumatologists from both primary and secondary care services is a significant factor contributing to the delay in diagnosis. In one study, it was discovered that there was an average wait of around 10 months from the initial report of back pain in a non-rheumatology setting to being referred to a rheumatologist. However, after the referral, the diagnosis of AS took only about one month [15].

Limited referrals occur due to misconceptions about axSpA, where it is incorrectly seen as a predominantly male disease, and there is a belief that elevated inflammatory markers are essential for diagnosis [16]. Furthermore, axSpA is an uncommon cause of a common symptom (back pain) which lacks pathognomonic symptoms and signs, increasing the difficulty in identifying patients who require referral to a rheumatologist [16]. The lack of validated axSpA diagnostic criteria is also a contributing factor [16-19].

A prospective controlled study conducted across multiple centres showed that targeted training in SpA significantly increased a GP’s inclination to refer patients displaying symptoms of axial and peripheral SpA by over 40% [20]. This demonstrates that improving education in primary GP settings as well as secondary dermatology services in IBP recognition can increase onward referrals for patients who require it.

Notably, within our study, 17% of patients experiencing back pain for over three months did not seek medical advice despite their ongoing symptoms. There is a valuable opportunity to educate high-risk individuals, raising their awareness, and thereby encouraging them to pursue diagnosis promptly. This initiative could lead to early detection and timely treatment of the condition.

In addition, creating straightforward and easily manageable screening questionnaires for non-rheumatologists to recognise patients with IBP, coupled with streamlined referral pathways, could significantly boost the number of appropriate referrals to rheumatologists. Proft et al., who utilized a screening tool to refer 335 patients enduring chronic back pain lasting more than three months to a rheumatologist, found that among 100 consecutive patients evaluated, 14% received a confirmed diagnosis of axPsA [14]. Their study indicates that a user-friendly, dermatologist-centred screening tool could efficiently identify axPsA in individuals with psoriasis, making it a viable option for routine use in clinical settings.

Limitations

Our study utilized the Berlin Criteria with 81.2% sensitivity when meeting at least two criteria to identify patients with IBP [12]. As such, patients meeting these criteria should also undergo an assessment by a rheumatologist to confirm an axPsA diagnosis. This was not the primary objective of our study and thus we have not included this final axPsA diagnostic data, but we recognise this as a study limitation. Finally, our study’s small sample size was also a limiting factor.

## Conclusions

This study has demonstrated that IBP was common in patients with psoriasis; however, it was underdiagnosed in these patients. Dermatologists are in a strategic position to play a crucial role in the early detection of axSpA and its treatment. However, limited awareness of IBP hinders its identification in psoriasis patients and subsequent referral to rheumatologists. Educating dermatologists, primary care doctors, allied healthcare professionals as well as patients can improve axSpA recognition and prompt referral, ensuring timely care. The development of simple and easy-to-administer screening questionnaires to aid non-rheumatologists in identifying patients with IBP along with simplified referral pathways would also increase onward referrals of appropriate patients to rheumatologists. Additionally, fostering collaboration between dermatologists and rheumatologists to establish effective internal referral pathways and joint clinics will enhance the process of referring patients to rheumatology services.

## Appendices

Psoriasis survey questionnaire

We would be grateful if you could answer the following questions, which will take you around 2-3 minutes.

Required

1. Have you been diagnosed with inflammatory back pain/ankylosing spondylitis/axial spondyloarthritis?

Yes

No

2. D.O.B?

3. Gender?

Male

Female

Other

Prefer not to say

4. How long (in years) have you had psoriasis for?

5. Are you currently taking biologic medication?

Yes

No

6. If yes - which biologic are you currently taking?

Adalimumab (Humira/Amgevita/Hyrimoz/Imraldi)

Certolizumab pegol (Cimzia)

Secukinumab (Consentyx)

Tildrakizumab (Ilumya)

Brodalumab (Siliq)

Ixekizumab (Taltz)

Guselkumab (Tremfya)

Infliximab (Remicade)

Bimekizumab (Bimzelx)

Ustekinumab (Stelara)

golimumab (Simponi)

7. If no - have you ever taken biologic medication? (Select all relevant)

Adalimumab (Humira/Amgevita/Hyrimoz/Imraldi)

Certolizumab pegol (Cimzia)

Secukinumab (Consentyx)

Tildrakizumab (Ilumya)

Brodalumab (Siliq)

Ixekizumab (Taltz)

Guselkumab (Tremfya)

Infliximab (Remicade)

Bimekizumab (Bimzelx)

Ustekinumab (Stelara)

golimumab (Simponi)

8. Do you experience back pain that has lasted for longer than 3 months?

Yes

No

9. If yes - does it improve with NSAIDs, i.e., ibuprofen, Naproxen?

Yes

No

10. How old were you (in years) when your back pain started?

11. Where is your pain located? (Select all relevant)

Neck

Mid back

Lower back

N/A

12. Does your back pain improve with activity or rest?

Activity

Rest

Neither

N/A

13. Have you ever seen a healthcare professional regarding your lower back pain?

Yes

No

14. If yes - which healthcare professionals have you seen? (Select all relevant)

GP

Physiotherapist

Chiropractor/Osteopath

Orthopaedic/Neurosurgeon

Rheumatologist

15. Do you experience morning stiffness lasting longer than 30 minutes?

Yes

No

16. Do you wake during the second half of the night due to back pain?

Yes

No

17. Do you have alternating buttock pain?

Yes

No

18. Do you experience pain and swelling in other joints?

Yes

No

19. Have you ever had plantar fasciitis (pain in the heel/arch of your foot) or Achilles tendinitis (pain in the tendon at the back of your ankle)?

Yes

No

20. Have you ever had uveitis/eye inflammation/red eye?

Yes

No

21. Have you ever had inflammatory bowel disease?

Yes

No

22. Does anyone in your family have ankylosing spondylitis?

Yes

No

## References

[1] Zhao SS, Pittam B, Harrison NL, Ahmed AE, Goodson NJ, Hughes DM (2021). Diagnostic delay in axial spondyloarthritis: a systematic review and meta-analysis. Rheumatology (Oxford).

[2] Seo MR, Baek HL, Yoon HH, Ryu HJ, Choi HJ, Baek HJ, Ko KP (2015). Delayed diagnosis is linked to worse outcomes and unfavourable treatment responses in patients with axial spondyloarthritis. Clin Rheumatol.

[3] Feld J, Chandran V, Haroon N, Inman R, Gladman D (2018). Axial disease in psoriatic arthritis and ankylosing spondylitis: a critical comparison. Nat Rev Rheumatol.

[4] Alinaghi F, Calov M, Kristensen LE (2019). Prevalence of psoriatic arthritis in patients with psoriasis: a systematic review and meta-analysis of observational and clinical studies. J Am Acad Dermatol.

[5] Mease PJ, Gladman DD, Papp KA (2013). Prevalence of rheumatologist-diagnosed psoriatic arthritis in patients with psoriasis in European/North American dermatology clinics. J Am Acad Dermatol.

[6] Gladman DD (2007). Axial disease in psoriatic arthritis. Curr Rheumatol Rep.

[7] Mease PJ, Palmer JB, Liu M (2018). Influence of axial involvement on clinical characteristics of psoriatic arthritis: analysis from the Corrona Psoriatic Arthritis/Spondyloarthritis Registry. J Rheumatol.

[8] Siebert S, Sengupta R, Alexander T (2016). Extra-articular manifestations of axial spondyloarthritis. Axial Spondyloarthritis.

[9] Gottlieb AB, Merola JF (2021). Axial psoriatic arthritis: an update for dermatologists. J Am Acad Dermatol.

[10] (2023). Axial SpA - How long are patients waiting for a diagnosis?. https://nass.co.uk/homepage/health-professionals/aspiring-to-excellence/axial-spa-how-long-are-patients-waiting-for-a-diagnosis-read-our-first-report-from-the-nass-time-to-diagnosis-audit/.

[11] Bamford S, Tahir H, Ladan Z, Hanumunthadu D (2023). Patient survey exploring the burden of inflammatory back pain in patients with uveitis. Cureus.

[12] Rudwaleit M, Metter A, Listing J, Sieper J, Braun J (2006). Inflammatory back pain in ankylosing spondylitis: a reassessment of the clinical history for application as classification and diagnostic criteria. Arthritis Rheum.

[13] Haroon M, Kirby B, FitzGerald O (2013). High prevalence of psoriatic arthritis in patients with severe psoriasis with suboptimal performance of screening questionnaires. Ann Rheum Dis.

[14] Proft F, Lüders S, Hunter T (2022). Early identification of axial psoriatic arthritis among patients with psoriasis: a prospective multicentre study. Ann Rheum Dis.

[15] Deodhar A, Mittal M, Reilly P, Bao Y, Manthena S, Anderson J, Joshi A (2016). Ankylosing spondylitis diagnosis in US patients with back pain: identifying providers involved and factors associated with rheumatology referral delay. Clin Rheumatol.

[16] Barnett R, Ingram T, Sengupta R (2020). Axial spondyloarthritis 10 years on: still looking for the lost tribe. Rheumatology (Oxford).

[17] Sykes MP, Doll H, Sengupta R, Gaffney K (2015). Delay to diagnosis in axial spondyloarthritis: are we improving in the UK?. Rheumatology (Oxford).

[18] Redeker I, Callhoff J, Hoffmann F, Haibel H, Sieper J, Zink A, Poddubnyy D (2019). Determinants of diagnostic delay in axial spondyloarthritis: an analysis based on linked claims and patient-reported survey data. Rheumatology (Oxford).

[19] Gaffney K, Webb D, Sengupta R (2021). Delayed diagnosis in axial spondyloarthritis-how can we do better?. Rheumatology (Oxford).

[20] van Onna M, Gorter S, Maiburg B, Waagenaar G, van Tubergen A (2015). Education improves referral of patients suspected of having spondyloarthritis by general practitioners: a study with unannounced standardised patients in daily practice. RMD Open.

